# On the Use of Impure Gold for Dental Purposes

**Published:** 1851-10

**Authors:** W. Imrie

**Affiliations:** Surgeon Dentist.


[*London Lancet.*

### ARTICLE XVII.

*On the Use of Impure Gold for Dental Purposes.* By W. Imrie, Surgeon Dentist.

During the last few months the public have been considerably alarmed by published statements to the effect that the gold used by cheap dentists is so largely alloyed with copper and other metals as seriously to injure the health of persons wearing artificial teeth. It has been declared that numerous persons have, after the introduction of artificial teeth into the mouth, been subject to all the symptoms of slow poisoning by deleterious salts of copper, produced by the action of the saliva on the plates upon which teeth have been mounted. It is of very great importance both for the satisfaction of the public and the credit of respectable dentists, that this matter should be rigidly inquired into. From the facts which have been made known, it seems impossible to doubt that many persons have been injured in health by wearing impure metallic plates. The writer has known one person, a medical practitioner, who, after wearing a plate for some months, which, on examination, was proved to consist chiefly of copper, suffered greatly from irritation of the stomach and bowels, with considerable emaciation, and great irritability and prostration of the nervous system. This gentleman commenced an action at law against the dentist, and it was only interrupted by his death from another cause.

The gold generally used by respectable dentists in this country is eighteen or twenty carat gold. Gold of eighteen carats is composed of eighteen parts of gold, and six parts of alloy; the alloy being formed either of two parts of copper and four of silver, or of two parts of silver and four of copper, according to the degree of hardness required. In eighteen carat gold there would thus always be either two or four parts out of twenty-four consisting of copper. This proportion of alloy is necessary to impart strength and hardness to the gold used.

There is no doubt that cheap dentists use gold for making plates, springs, &c., for mounting teeth, alloyed to a far greater extent than eighteen carat gold, so that the alloy equals or even exceeds the gold in quantity. Indeed, the metal used in some cases, is so base, that the plates require to be gilt to impose on the parties wearing them. Of course the gilding, after a time, wears off, when the deleterious alloy is fully and constantly exposed to the chemical action of the saliva.

I have been so much interested in the important questions arising out of these facts, that I have taken the opinion of my friend and patient, Dr. Taylor, the distinguished analytical chemist, of Guy's hospital, respecting them. I proposed to Dr. Taylor the questions which suggested themselves to me, and I have received from him the following statement in reply, which is highly important, both on account of the interest which attaches to the subject itself, and from the unquestionable authority of Dr. Taylor:

*Action of the Saliva on the Gold or Gilt Plates used in Dentistry.*—If copper alone were used, a sub-chloride of the metal would undoubtedly be formed by the action of the chloride of sodima (salt) in the saliva on the copper. There would be a well marked brassy or coppery taste. The mucus of the saliva is generally acid, sometimes highly so. Under these circumstances, from the free access of oxygen by the mouth, the copper would be in part oxydized, and the oxyd thus formed would combine with the albumen and mucus, and be carried into the stomach, where the effect would be, in the course of time, that of slow poisoning by copper—griping colicky pains in the bowels, occasional purging, emaciation, loss of appetite, salivation (saliva greenish colored,) and in continued cases even paralysis. Copper has a tendency to accumulate, and thus produce bad effects. I do not, however, think that any of these symptoms could occur to a dangerous extent without the patient being thoroughly warned by the disagreeable metallic taste in the mouth. When copper is alloyed with gold and silver, a galvanic effect is produced by contact with the saliva, by which the chemical action on the copper is increased. There is no chemical action on the gold, and the effect on the silver is chiefly to darken it by producing a sulphuret from the sulphur contained in the salts of the saliva and the mucus and albumen (ptyalin.) The saliva does not produce with the alloy of gold and silver any compounds which can produce injury to health. From what has been said, therefore, it follows that unless the copper be well protected by a large admixture of gold and silver (especially the former,) and thoroughly alloyed therewith, a noxious chemical action will be set up, likely in the course of time to produce effects injurious to health. The maximum quantity of copper contained in the gold of 18 carats, used by respectable dentists, is 16 per cent., while the gold is 75 per cent., and the silver about 9. I do not think that any alloy of this kind could have any effect injurious to health. Supposing the metals to be well alloyed, the copper would be, I believe, effectually protected. When the copper amounts to 50 per cent. or more, or the surface is merely coated with gold, then the effects of chronic poisoning by copper may be expected to take place. A. S. TAYLOR, M. D., F. R. S.

3, Cambridge-place, Regent's Park.

It will be seen that Dr. Taylor's observations go very decidedly to prove that the eighteen carat gold used by respectable dentists can have no deleterious action whatever upon the animal economy. It is equally evident from his remarks that a baser metal than this could not be used without danger to health, and even risk to life, if the quantity of copper in the mouth should be large. I have no doubt either, that in persons suffering from disordered health, the state of the saliva is so altered as to affect the copper plates more decidedly than would occur in persons in good health.

Various remedies have been suggested for the prevention of these mischiefs, by forcing dentists to use in their work, gold of sufficient purity to remove all risk of poisoning or injury. In particular, it has been proposed, that all gold plates, pivots, springs, &c., shall, after their manufacture, be sent to Goldsmith's Hall, there to be tested and marked, but I would submit, that with the present method of stamping, this proceeding would be impracticable. It is necessary for the ease and comfort of persons wearing artificial teeth, that the adaptation of the plate to the gums and palate should be perfect. Stamping the plates after their completion could not fail to injure them, and in cases requiring springs, these parts could not be stamped at all without destroying them. It would be difficult to suggest any other mode of marking gold, which should be a warranty of its being within the sufficient degree of purity.

In some cases ivory is used instead of gold for dental plates, as for instance in elderly persons in whom the alveolar ridges are sunken and absorbed; but in the great majority of cases ivory can never be a substitute for gold, owing to the incompatibility of a due degree of thinness and strength, in ivory plates.

The only remedies against the imposition of base metal upon the public, instead of gold of the requisite purity, seems to be, that persons shall be informed of the ordinary signs of inferior gold in the plates they wear. In suspicious cases, it would be easy by applying to a dentist of character, to have a small portion of the plate analysed, and if it were found sufficiently alloyed with copper or any other inferior metal to injure health, no doubt the parties thus offending could, and ought to be, legally punished.

Whenever the plates upon which sets, or partial sets, of artificial teeth are mounted become blackened and discolored by the action of saliva, and particularly when the discoloration is accompanied by a constant metallic taste, as of copper, the purity of the metal should be suspected, and the plate at once examined either by a practiced dentist or by an analytical chemist.

The public have, however, the great remedy in their own hands, which is, never to employ persons advertising themselves in the newspapers, or at the corners of the streets as cheap dentists—who profess to supply teeth in partial, or even full sets, at a price at which it is utterly impossible gold of the requisite purity for health can be used. Those persons are either willingly, or from mere carelessness deceived, who pay for an excessively cheap material, and expect a valuable article in return. If cases in which teeth have been mounted on impure gold are inquired into, it will be found, that they are instances in which cheap dentists have been employed. No man of station, and with a creditable reputation in dentistry, would dream of risking his reputation and practice by inserting poison in the mouths of his patients. I may repeat, then, that if bad dentistry be a great evil, the public have undoubtedly the remedy in their own hands.—*London Lancet.*

